# Metabolic diversity in sorghum: Mechanism underlying grain color variation and fermentation quality

**DOI:** 10.1371/journal.pone.0331980

**Published:** 2025-09-25

**Authors:** Fan Yang, Jiaqi Qiao, Miao Yang, Dongye Liu, Huimei Sun, Xinxin Wang, Xiao Zhang, Dongao Huo

**Affiliations:** College of Biological Sciences and Technology, Taiyuan Normal University, Jinzhong, China; China Three Gorges University, CHINA

## Abstract

Sorghum (*Sorghum bicolor* L. Moench) serves as a critical staple cereal, forage crop, and primary raw material for baijiu (Chinese distilled spirits) production and vinegar fermentation. In this study, we performed comprehensive untargeted metabolomic profiling on widely cultivated sorghum accessions exhibiting diverse grain color phenotypes, followed by in-depth characterization of their metabolic signatures. The results demonstrated significant inter-accession variability in metabolite composition, with GL18 showing the most pronounced accumulation of metabolites within the phenylpropanoids and polyketides class. KEGG pathway enrichment analysis revealed substantial divergence in flavonoid biosynthetic pathways among accessions, particularly in the biosynthesis of naringenin, delphinidin, cyanidin, and pelargonidin 3-glucoside—key pigments correlated with grain color variations. Metabolite profiling further identified distinct accumulation patterns of flavor precursors (e.g., β-phenylethanol precursors), amino acids (e.g., tryptophan, L-leucine), and glycosides (e.g., L-rhamnofuranose) that significantly influence baijiu sensory quality attributes. For vinegar fermentation, significant inter-accession differences were observed in carbohydrate (sucrose, mannitol), amino acid (L-proline, arginine), and organic acid (lactic acid, quinic acid) accumulation profiles, which correlated with fermentation efficiency and final product quality. This study provides novel insights into the metabolic basis of sorghum grain color diversity and highlights the potential for tailored sorghum accessions to enhance the quality and diversity of baijiu and vinegar products, thereby contributing to the optimization of crop quality and agricultural resource efficiency.

## Introduction

Sorghum (*S. bicolor*), a widely cultivated staple cereal, has been domesticated and utilized in China for millennia [1–4]. This crop serves as a key raw material in traditional Chinese fermentation industries, particularly for the production of baijiu (distilled spirits) and vinegar [5–8]. Baijiu produced from sorghum exhibits superior flavor profiles compared to other cereal-based spirits, as evidenced by studies demonstrating enhanced sensory attributes in sorghum-derived products [9]. The geographical origin and varietal characteristics of sorghum significantly influence baijiu flavor composition. Non-targeted metabolomic analyses revealed distinct metabolic signatures between sorghum accessions, including differential accumulation of sphingolipids, 2,5-diketopiperazines, and methionine derivatives [10]. Volatile compound profiling further demonstrated that varietal differences account for significant variations in baijiu aroma constituents, underscoring the critical role of sorghum variety selection in flavor modulation [11]. Similarly, vinegar fermentation exhibits strong material-dependent characteristics. Cereal-based vinegars derive their diverse volatile profiles from raw material composition, with sorghum contributing unique metabolic contributions [12–14]. Comparative analysis of sweet sorghum vinegar identified variety-specific compounds such as aconitic acid, along with elevated citric and isocitric acid levels compared to commercial vinegars [15]. During fermentation, sorghum promotes the formation of aromatic aldehydes (e.g., benzaldehyde, vanillin, 3,5-dihydroxybenzaldehyde), which significantly enhance vinegar aroma complexity [16]. These findings collectively establish that sorghum varietal differences constitute a primary determinant of flavor quality in both baijiu and vinegar production systems.

Grain color represents a key phenotypic trait in sorghum, exhibiting a broad spectrum from white to black with intermediate yellow and red [17]. These colored accessions serve as valuable germplasm resource, with each possessing phytochemical compositions and nutritional profiles. Generally, dark-colored seeds tend to contain higher level of nutrients such as polyphenols and flavonoids [18]. Multi-omics analyses have elucidated the molecular basis of grain color variation in sweet sorghum. Integrated metabolomic-transcriptomic profiling of three sweet sorghum accessions identified six flavonoid compounds—xanthohumol, naringin, prunetin, naringenin, hesperetin, and pinocembrin—as primary determinants of color variation [19]. Similarly, targeted metabolomics revealed significant accumulation of cyanidin derivatives in red sorghum seeds, suggesting their central role in anthocyanin-based coloration [20]. Comparative studies in quinoa (*Chenopodium quinoa*) further corroborated these findings, showing higher procyanidin B3/B2 levels in red/black accessions compared to white/yellow accessions [21]. Analogous observations in wheat (*Triticum aestivum*) demonstrated differential regulation of flavonoid biosynthetic pathways between purple and white grains [22]. These evidence establish flavonoid composition as the principal determinant of cereal grain color diversity. The metabolic pathways governing pigment accumulation not only explain phenotypic variation but also provide critical targets for breeding programs aimed at enhancing nutritional quality and functional properties. However, the mechanisms regulating flavonoid biosynthesis remain poorly characterized, particularly in sorghum germplasm with contrasting grain color phenotypes.

Metabolomics has emerged as a systemic research approach, enabling comprehensive characterization of metabolic variations in biological systems and facilitating crop improvement [23]. This study conducted untargeted metabolomic profiling on three sorghum accessions exhibiting distinct grain color phenotypes (black, red, and white). Through comparative analysis of their metabolic profiles, we identified key flavonoid compounds responsible for color differentiation. We also evaluated the impact of metabolite accumulation profiles on the quality attributes of baijiu and vinegar fermentation. These findings advance our understanding of sorghum metabolic diversity while providing a mechanistic framework for optimizing crop utilization in food fermentation processes.

## Materials and methods

### Plant materials

Eleven sorghum accessions with different grain colors were selected for this study, designated as GL1, GL5, GL18, GL21, GL62, GL74, GL171, GL179, GL196, GL200, and GL540. These materials were provided by the Center for Agricultural Genetic Resources Research at Shanxi Agricultural University. These materials were sown on April 16, 2022 and cultivated under uniform field conditions at the Dongyang experimental station of Taiyuan Normal University in Jinzhong (36°85’ N, 111°77’ E), Shanxi province, China. During the growing season, average daily temperature ranged between 15–28°C with 8–10 h daily sunshine. The soil was a neutral loam (pH 6.5–7.5) containing 2–3% organic matter. Despite the semi-arid climate, soil moisture was maintained through irrigation. A randomized block design with three replications was employed. Each plot consisted of 15 rows. At the flowering stage, panicles were bagged to prevent cross-pollination. Upon maturity, grains from each accession were harvested.

### Gene expression analysis

For *SbCHS1*, *SbCHS2*, and *SbANS1* expression analysis, sorghum grains were collected, immediately frozen in liquid nitrogen, and stored at −80°C for RNA extraction. Total RNA was extracted from three biological replicates, with each consisting of a pooled sample of five plants. Total RNA was extracted from tissues using the RNeasy plant mini kit (QIAGEN). The isolated RNA was reverse transcribed using SuperScript III reverse transcriptase (Invitrogen). Quantitative RT-PCR (RT-qPCR) was performed utilizing SYBR Green Real-Time PCR Master Mixes (Invitrogen).

### Untargeted metabolomics analysis of sorghum samples

#### Sample extraction and preparation.

The collected sorghum samples were lyophilized using a freeze-dryer (Alpha 1–2LD plus, Christ, Germany) until complete desiccation. The dried grains were then transferred into 2 mL centrifuge tubes and homogenized into a fine powder using a ball mill (BM 500, Anton Paar, Austria). For each sample, 0.3 mg of powdered grain was precisely weighed and transferred into individual 2 mL centrifuge tubes, with three replicates prepared per sample. To each tube, 0.6 mL methanol containing internal standards (10 μg/mL Succinic acid-2, 2, 3, 3-d4, 15 μg/mL Cholic acid-2, 2, 3, 4, 4-d5, 5 μg/mL DL-Tryptophan-2, 3, 3-d3, 10 μg/mL DL-Methionine-3, 3, 4, 4-d4, 5 μg/mL L-Phenylalanine (ring-d5) and 5 μg/mL Choline chloride (trimethyl-d9)) was precisely added. The mixture was vortexed for 1 min, followed by ultrasonication at room temperature for 15 min. The samples were then centrifuged at 12,000 rpm for 10 min at 4°C using a centrifuge (H1850R, Xiangyi, China). The supernatant was filtered through a 0.22 μm membrane, and the filtrate was transferred into vials for subsequent LC-MS analysis.

#### Liquid chromatography conditions.

Chromatographic separation was performed using a Thermo Vanquish ultra-high-performance liquid chromatography (UHPLC) system (Thermo Fisher Scientific, USA) equipped with an ACQUITY UPLC® HSS T3 column (2.1 × 150 mm, 1.8 µm; Waters, Milford, MA, USA). The flow rate was maintained at 0.25 mL/min, with a column temperature of 40°C and an injection volume of 2 μL. For positive ion mode, the mobile phase consisted of 0.1% formic acid in acetonitrile (B2) and 0.1% formic acid in water (A2). The gradient elution program was as follows: 0–1 min, 2% B2; 1–9 min, 2% − 50% B2; 9–12 min, 50% − 98% B2; 12–13.5 min, 98% B2; 13.5–14 min, 98% − 2% B2; 14–20 min, 2% B2. For negative ion mode, the mobile phase consisted of acetonitrile (B3) and 5 mM ammonium formate in water (A3). The gradient elution program was as follows: 0–1 min, 2% B3; 1–9 min, 2% − 50% B3; 9–12 min, 50% − 98% B3; 12–13.5 min, 98% B3; 13.5–14 min, 98% − 2% B3; 14–17 min, 2% B3.

#### Mass spectrometry conditions.

Mass spectrometry analysis was conducted using a Thermo Orbitrap Exploris 120 mass spectrometer (Thermo Fisher Scientific, USA) equipped with an electrospray ionization (ESI) source. Data were acquired in both positive and negative ion modes. The spray voltage was set to 3.50 kV for positive ion mode and −2.50 kV for negative ion mode. The sheath gas and auxiliary gas flow rates were set to 30 arb and 10 arb, respectively. The capillary temperature was maintained at 325°C. Full-scan MS spectra were acquired at a resolution of 60,000, with a scan range of m/z 100–1000. Higher-energy collisional dissociation (HCD) was employed for MS/MS fragmentation with a collision energy of 30%. MS/MS spectra were acquired at a resolution of 15,000, with the top 4 ions selected for fragmentation. Dynamic exclusion was applied to minimize redundant MS/MS acquisitions.

### Data processing

Data analysis, including principal component analysis (PCA), KEGG enrichment analysis, hierarchical clustering heatmaps, and cluster analysis, was performed on the BioDeep cloud platform (https://v2.biodeep.cn/home/tool). Statistical analysis and variance analysis were conducted using SPSS 24.0. Visualization of data, including line charts, boxplots, bar charts, and cluster plots, was performed using R 4.3.1.

### Field site access statement

Field site did not require specific permits because: The experimental site is an institutional research station managed by Taiyuan Normal University, and it is primarily used for academic research and teaching purposes. No protected areas or endangered species were involved, complying with Environmental Protection Law of China.

## Results

### Untargeted metabolomics analysis in sorghum accessions

**Fig 1:**
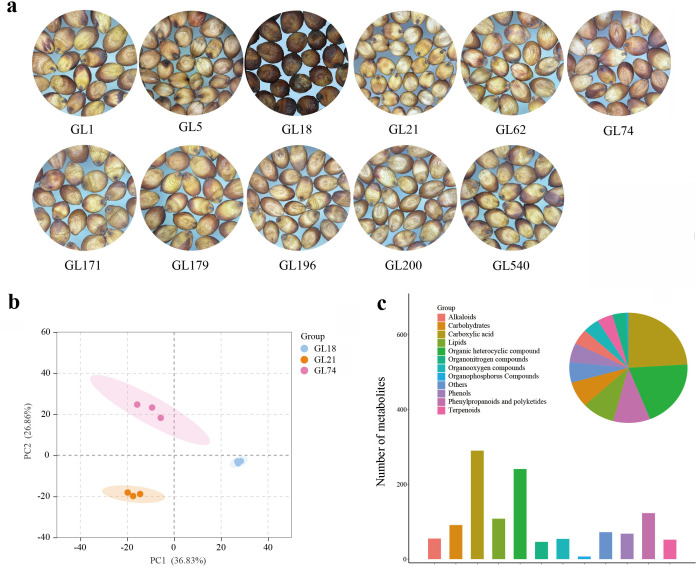
Metabolomic data analysis in sorghum accessions. (a) Mature grain images of every sorghum accession. (b) Principal component analysis (PCA) of untargeted metabolomics data in sorghum accessions. (c) Metabolites classification in sorghum accessions.

Three representative sorghum accessions with different grain colors, including GL18 (black grain), GL21 (yellow), and GL74 (red) were selected for this study (Fig 1a). Untargeted metabolomics was employed to systematically investigate the metabolic differences across sorghum accessions. Principal component analysis (PCA) was performed on the metabolomics profiles in sorghum accessions. The first two principal components collectively accounted for 63.69% of the total variance, with PC1 contributing 36.83% and PC2 contributing 26.86%. Notably, the three biological replicates for each sorghum accession clustered tightly, indicating minimal intra-varietal variability and high experimental reproducibility. Furthermore, these sorghum accessions exhibited clear separation in PCA plot, which reflected significant differences in metabolite accumulation patterns among the accessions (Fig 1b). A total of 1,027 metabolites were identified and classified into 12 distinct categories. Among these, carboxylic acids constituted the largest proportion (24.03%, 290 metabolites), followed by organic heterocyclic compounds (19.97%, 241 metabolites) and phenylpropanoids and polyketides (10.19%, 123 metabolites), collectively representing the most abundant metabolite classes. Other notable categories included lipids (8.95%), carbohydrates (7.54%), phenolic compounds (5.97%), alkaloids (5.63%), and organic oxygen compounds (4.56%) (Fig 1c).

### Analysis of differentially accumulated metabolites among sorghum accessions

**Fig 2:**
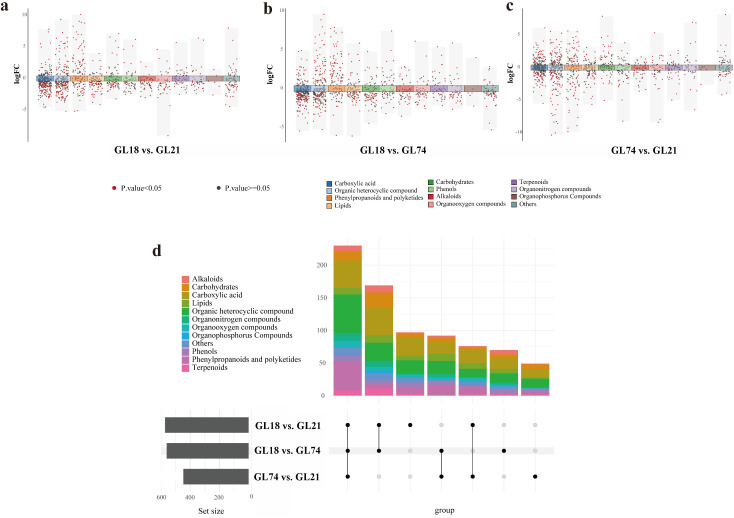
Analysis of DAMs among sorghum accessions. (a) Classification and quantification of DAMs between GL18 and GL21. (b) Classification and quantification of DAMs between GL18 and GL74. (c) Classification and quantification of DAMs between GL74 and GL21. The x-axis represents primary metabolite categories, while the y-axis denotes the log-transformed fold change values. (d) Upset analysis illustrating the overlap and uniqueness of DAMs across the three pairwise comparisons.

Using strict selection criteria (Variable Importance in Projection [VIP] > 1, fold change > 2, and *P*-value < 0.05), we performed a comprehensive analysis of differentially accumulated metabolites (DAMs) across sorghum accessions: GL18, GL21, and GL74 to elucidate their metabolic disparities. Pairwise comparisons revealed distinct DAM profiles: in GL18 vs. GL21 analysis, 572 DAMs were identified, of which 157 were upregulated and 415 were downregulated in GL18 relative to GL21(Fig 2a); GL18 vs. GL74 comparison yielded 561 DAMs, with 208 upregulated and 353 downregulated (Fig 2b), while GL74 vs. GL21 comparison detected 447 DAMs, including 209 upregulated and 238 downregulated (Fig 2c). Class-specific analysis demonstrated that phenylpropanoids and polyketides exhibited the highest number of upregulated DAMs in GL18 compared to both GL21 and GL74. GL74 vs. GL21 comparison revealed that carboxylic acids constituted the most abundant class among upregulated DAMs, whereas organic heterocyclic compounds predominated among downregulated DAMs (Fig 2).

Upset analysis of DAMs across all pairwise comparisons revealed that 97, 70, and 49 DAMs were uniquely identified in GL18 vs. GL21, GL18 vs. GL74, and GL74 vs. GL21 comparisons, respectively. Additionally, 230 DAMs were shared among all three pairwise comparisons. Among these shared metabolites, organic heterocyclic compounds constituted the most abundant class, followed by phenylpropanoids and polyketides (Fig 2d).

### KEGG pathway enrichment analysis of DAMs across sorghum accessions

The DAMs identified in the pairwise comparisons of GL18 vs. GL21, GL18 vs. GL74, and GL74 vs. GL21 were mapped to the KEGG database, and their distribution across metabolic pathways was systematically analyzed (S1 Table). KEGG pathway enrichment analysis revealed 14 significantly enriched pathways in GL18 vs. GL21 comparison, including five secondary metabolic pathways. Similarly, 12 significantly enriched pathways were identified in GL18 vs. GL74 comparison, with four secondary metabolic pathways among them. In GL74 vs. GL21 comparison, eight significantly enriched pathways were detected, half of which were secondary metabolic pathways (*P* < 0.05). Notably, in all pairwise comparisons among these sorghum accessions, DAMs mapped to flavonoid biosynthesis pathways (including flavonoid, flavone/flavanol, and isoflavonoid biosynthesis) accounted for over 20% of total metabolites identified in each respective pathway. This finding underscores the distinct metabolic profiles of GL18, GL21, and GL74 in flavonoid-related secondary metabolism. The significant changes in flavonoid profiles and their enrichment in secondary metabolic pathways may directly or indirectly influence grain color. We focused on the variations in flavonoid compounds and their associated metabolic pathways, aiming to uncover the molecular mechanisms underlying color variation in sorghum grains.

### Differential accumulation patterns of flavonoid metabolites across sorghum accessions

**Fig 3:**
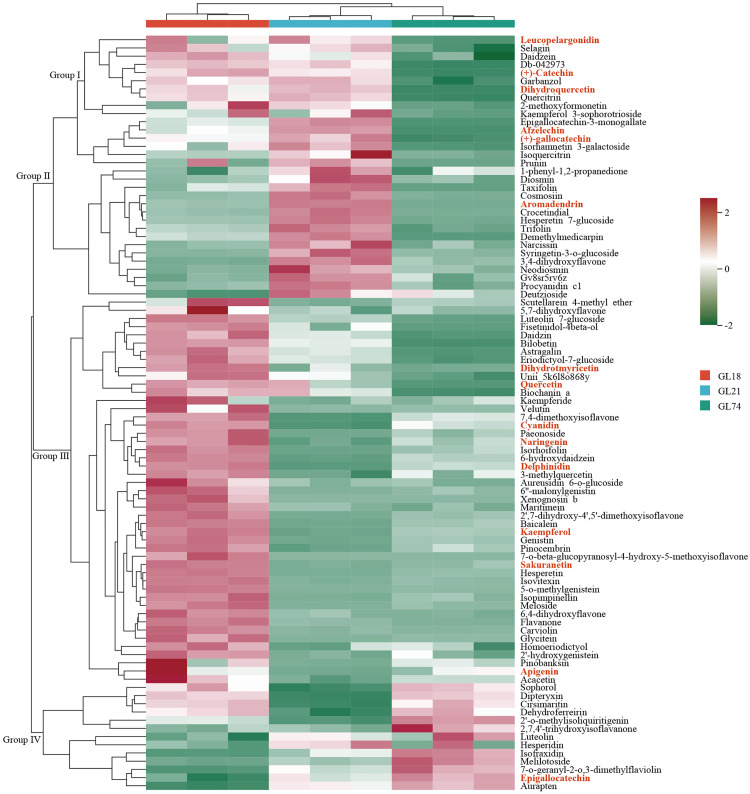
Heatmap of differentially accumulated flavonoid metabolites across sorghum accessions. The x-axis represents different accessions, while the y-axis lists the identified metabolites. Metabolites associated with sorghum grain color are highlighted in red for clarity. The color gradient represents relative abundance level.

A comprehensive metabolomic profiling of flavonoid metabolites was conducted across sorghum accessions, identifying a total of 123 differentially accumulated flavonoid metabolites. Based on stringent selection criteria (VIP > 1.0, fold change > 2.0, and *P*-value < 0.05), pairwise comparisons revealed distinct accumulation patterns: in GL18 vs. GL21 comparison, 72 differentially accumulated flavonoid metabolites exhibited significant differences, comprising 46 upregulated and 25 downregulated metabolites; while GL18 vs. GL74 comparison identified 68 differentially accumulated flavonoid metabolites, including 59 upregulated and 9 downregulated metabolites. GL74 vs. GL21 comparison showed 74 differentially accumulated flavonoid metabolites, with 36 upregulated and 37 downregulated metabolites. Notably, GL18 demonstrated consistently higher accumulation level for the majority of flavonoid metabolites compared to both GL21 and GL74, suggesting that GL18 possesses unique metabolic characteristics likely governed by accession-specific regulatory mechanisms in flavonoid biosynthesis pathways, which may contribute to its distinct phenotypic traits (Fig 3).

The relative abundance variations of all identified differentially accumulated flavonoid metabolites across sorghum accessions were visualized in Fig 3, where clustering analysis categorized these metabolites into four distinct metabolic groups. Group I comprised 10 metabolites exhibiting significantly higher accumulation in both GL18 and GL21 compared to GL74. Group II contained 22 metabolites with the highest level in GL21, followed by GL18, and markedly lower level in GL74. Group III consisted of 46 metabolites showing exclusive high accumulation in GL18 but significantly reduced level in both GL21 and GL74. Group IV included 13 metabolites with low abundance in GL18 and GL21 yet pronounced accumulation in GL74. The heatmap analysis reveals that GL18 demonstrates a distinct metabolic signature characterized by elevated flavonoid accumulation compared to GL21 and GL74, particularly for Group III metabolites. These findings provide compelling evidence for accession-specific regulatory mechanisms governing flavonoid biosynthesis, which likely contribute to the divergent grain color phenotypes observed among these sorghum accessions.

### Comparative analysis of color-related metabolites across flavonoid biosynthetic pathways

**Fig 4:**
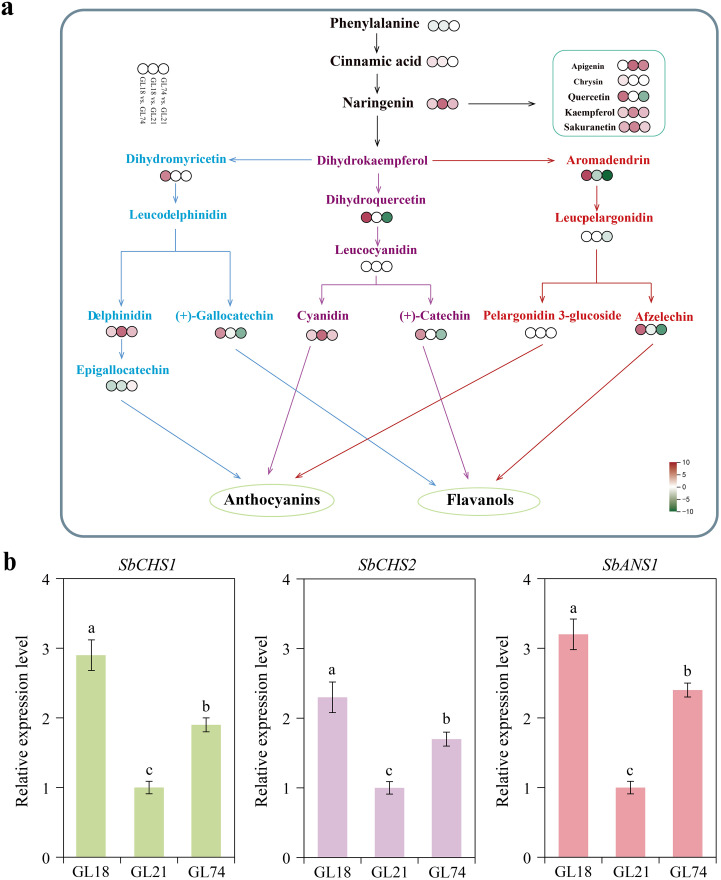
Analysis of flavonoid metabolic pathways in sorghum accessions. (a) Comparative visualization of flavonoid metabolic pathway variations across sorghum accessions. The color intensity within each circle indicates the fold change of metabolites in pairwise comparisons between accessions. (b) Transcript accumulation patterns of key flavonoid biosynthetic genes (*SbCHS1*, *SbCHS2*, and *SbANS1*) across sorghum accessions. Values represented means ± SE (n = 15). Different letters above columns indicated statistically significant differences (*LSD* test, *P* < 0.05).

Color-related flavonoid metabolites were systematically mapped onto the flavonoid biosynthetic pathway (Fig 4a), which initiates with phenylpropanoid pathway conversion of phenylalanine to naringenin through chalcone synthase (CHS). Quantitative metabolite profiling revealed distinct accession-specific accumulation patterns: phenylalanine showed lower level in GL18 compared to GL21 and GL74, while its immediate product cinnamic acid accumulated higher in GL18. The downstream metabolite naringenin exhibited maximum accumulation in GL18, moderate level in GL74, and minimum level in GL21. This differential distribution suggests a potential metabolic bottleneck in GL21, where limited naringenin availability may constrain the downstream anthocyanin biosynthesis pathway. Such limitation in GL21 may correlate with its lighter grain color, while GL18’s elevated naringenin level likely support enhanced anthocyanin accumulation. Quantitative real-time PCR (qRT-PCR) analysis revealed that *SbCHS1* and *SbCHS2* exhibited highest expression level in GL18, while showing lowest expression in GL21 (Fig 4b). This transcriptional profile consistently matched the accumulation pattern of naringenin detected through metabolomic profiling.

Naringenin serves as a general precursor for anthocyanins, proanthocyanidins, and flavonoids [24] and can enter the anthocyanin metabolic pathway through three distinct pathways. In the first pathway (Fig 4a left), naringenin is converted to delphinidin, (+)-gallocatechin, and epigallocatechin via dihydromyricetin. Metabolites analysis revealed that, except for epigallocatechin, all metabolites in this pathway were more abundant in GL18. In GL21, the metabolic flux favored (+)-gallocatechin, while in GL74, it was biased toward epigallocatechin. Delphinidin, a precursor of blue-purple delphinidin derivatives, was inferred to be more abundant in GL18, whereas its production in GL21 appeared to be relatively limited. In the second pathway (Fig 4a middle), naringenin is metabolized to cyanidin and (+)-catechin through anthocyanin synthase (ANS). All metabolites in this pathway were highly abundant in GL18. In GL21, the metabolic flux was directed toward (+)-catechin. Cyanidin, the precursor of magenta-colored cyanidin derivatives, likely contributed to enhanced accumulation of these pigments in GL18 and GL74. In contrast, GL21 exhibited distinct metabolic characteristics due to the preferential accumulation of (+)-catechin, which redirected metabolic flux away from cyanidin biosynthesis. qRT-PCR analysis revealed that *SbANS1* exhibited highest expression level in GL18, while showing lowest expression in GL21 (Fig 4b). In the third pathway (Fig 4a right), naringenin is converted to pelargonidin 3-glucoside via aromadendrin. The level of pelargonidin 3-glucoside showed no significant differences among these accessions, indicating that this pathway may serve as a foundational metabolic route for color formation in all sorghum accessions. In summary, the flavonoid biosynthetic pathways exhibited significant variations among sorghum accessions. The first and second pathways may primarily modulate grain color in GL18 and GL74, while the third pathway provided a foundational metabolic basis for color formation across all sorghum accessions. These findings offer critical insights into the metabolic regulation mechanisms underlying sorghum grain color.

### Key metabolite accumulation pattern influencing baijiu and vinegar fermentation quality

**Fig 5:**
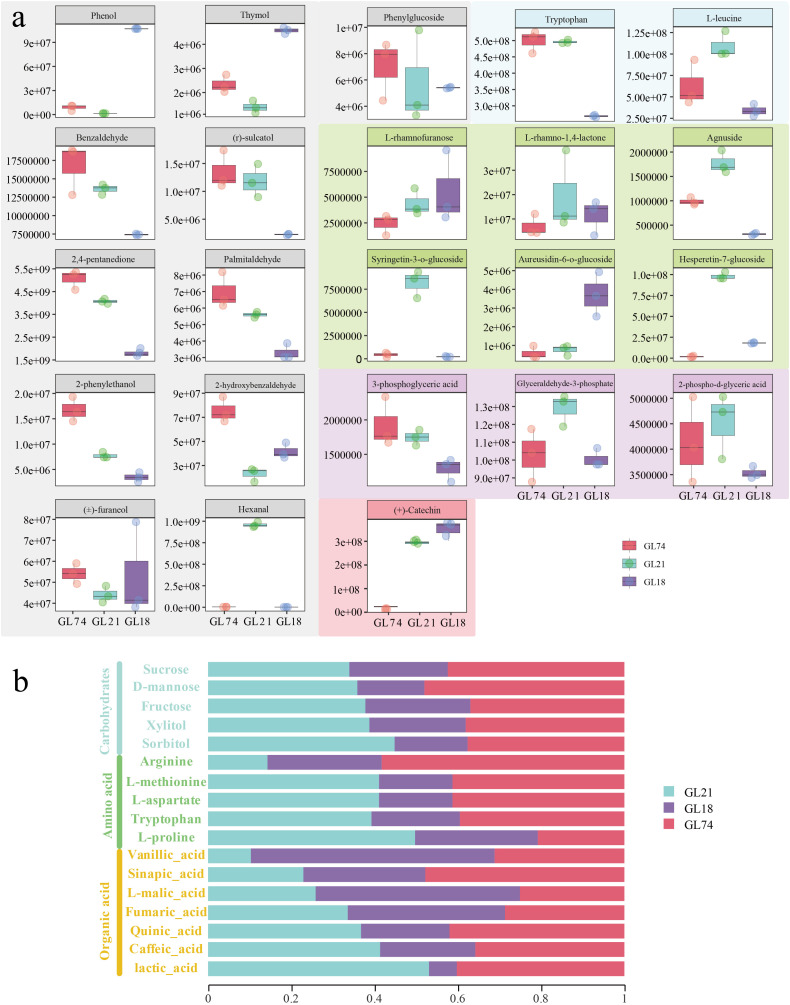
Metabolite profiling associated with baijiu and vinegar fermentation in sorghum accessions. (a) Baijiu fermentation-associated metabolites. The x-axis displays distinct sorghum accessions, while the y-axis indicates relative metabolite abundance. Metabolites are functionally categorized by colored boxes: gray-boxed compounds represent flavor-active metabolites during fermentation; blue-boxed compounds denote amino acid-derived metabolites; green-boxed compounds correspond to sugar-related metabolites; purple-boxed compounds indicate energy metabolism intermediates; and pink-boxed compounds represent condensed tannins. (b) Vinegar fermentation-related metabolite distribution. The x-axis shows the percentage contribution of each compound within an accession relative to its total accumulation level across all accessions. The y-axis lists compounds categorized into three functional groups: carbohydrates (blue), amino acid-derived metabolites (green), and organic acid-related metabolites (yellow).

Based on metabolomic profiling in sorghum accessions, we identified key metabolites associated with baijiu fermentation quality, including flavor-active compounds, amino acids, glycosides, and energy metabolism intermediates. Comparative analysis revealed distinct metabolic signatures among sorghum accessions: phenol and thymol exhibited significantly higher accumulation in GL18 compared to GL21 and GL74, whereas benzaldehyde, (R)-sulcatol, 2,4-pentanedione, and palmitaldehyde showed predominant enrichment in GL21 and GL74. Notably, 2-phenylethanol, 2-hydroxybenzaldehyde, and (±)-furaneol demonstrated notably elevated level specifically in GL74, with phenylglucoside—a critical precursor in β-phenylethanol biosynthesis—reaching higher accumulation level in GL74. In contrast, hexanal accumulation was greater in GL21 (Fig 5a). These metabolic distinctions may establish metabolite-fermentation quality relationships at the molecular level.

Amino acids such as tryptophan and L-leucine, which significantly influence baijiu quality, were detected at lower accumulation level in GL18 but at higher level in GL21 and GL74. Glycosides including L-rhamnofuranose, L-rhamno-1,4-lactone, agnuside, syringetin-3-O-glucoside, aureusidin 6-O-glucoside, and hesperetin 7-glucoside exhibited significantly greater accumulation in GL21 compared to the other two accessions. These glycosides function as flavor precursors during fermentation, indicating that GL21 may have enhanced potential for producing a broader spectrum of flavor compounds. Metabolic profiling also revealed differences in energy metabolism-related metabolites: 3-phosphoglyceric acid showed reduced level in GL18, whereas glyceraldehyde 3-phosphate and 2-phospho-D-glyceric acid were more abundant in GL21, suggesting comparatively active energy metabolism in GL21. Furthermore, (+)-catechin—a monomer of proanthocyanidins (condensed tannins)—displayed no significant difference between GL18 and GL21 but lower in GL74. Tannins contribute to the formation of aromatic compounds such as syringic acid and syringaldehyde during fermentation, thereby enhancing the aromatic complexity of baijiu. Consequently, GL18 and GL21 may serve as superior raw materials for baijiu production owing to their potential to yield more beneficial flavor compounds. In summary, the observed metabolic variations among sorghum accessions may significantly influence the flavor profile and quality characteristics of the resulting baijiu.

Furthermore, this study identified key metabolites associated with vinegar fermentation, including carbohydrates, amino acids, and organic acids. As shown in Fig 5b, the accumulation level of carbohydrates was significantly higher in GL21 and GL74 compared to GL18. Among amino acids, L-proline exhibited greater accumulation in GL21 but was present at relatively lower level in GL74, whereas arginine showed higher level in GL74. The level of other amino acids remained consistent across these accessions. For organic acids, vanillic acid and L-malic acid were more abundant in GL18, lactic acid and caffeic acid were predominant in GL21, and sinapic acid and quinic acid demonstrated higher accumulation in GL74. The distinct metabolite profiles observed among these sorghum accessions provide a critical foundation for subsequent vinegar fermentation processes.

## Discussion

### Accession-specific flavonoid pathway regulation shaping sorghum grain color

Flavonoids, recognized as pivotal secondary metabolites in plants [25], have been extensively documented to exhibit a strong correlation with grain coloration. Metabolomic analysis of yellow and black Brassica seeds revealed 116 differentially accumulated metabolites, with the majority being associated with the flavonoid biosynthetic pathway [26]. Similarly, comparative metabolomic profiling of mungbean seed coats demonstrated that the total anthocyanin accumulation level in black-coated seeds was significantly elevated compared to that in conventional varieties [27]. Furthermore, a study on the pigment composition of 10 differently colored adzuki bean seed coats indicated that anthocyanins are the primary pigments, while the combination of proanthocyanidins and anthocyanins significantly influences the diversity of seed coat coloration [28]. Yang et al. identified flavonoid biosynthesis and flavone and flavonol biosynthesis as significantly enriched pathways in differently colored highland barley through metabolomic analysis, further underscoring the critical role of flavonoids in seed color formation [18]. In this study, a total of 123 flavonoid metabolites were identified through untargeted metabolomic profiling. GL18 exhibited significantly higher total flavonoid accumulation compared to both GL21 and GL74. KEGG pathway enrichment analysis revealed inter-accession variation in flavonoid biosynthetic pathway, suggesting a potential regulatory role in grain color determination. These findings provide critical insights into the metabolic basis of phenotypic diversity among sorghum germplasm. Building on this metabolic characterization, subsequent analyses focused on dissecting the specific regulatory mechanisms of flavonoid biosynthesis in grain color formation.

The first step in flavonoid biosynthesis pathway involves the conversion of phenylalanine to naringenin via cinnamic acid [29]. Naringenin accumulation exhibited variation: GL18 showed the highest accumulation level, followed by GL74, while GL21 demonstrated the lowest accumulation. Chalcone synthase (CHS) and chalcone isomerase (CHI) play essential roles in flavonoid synthesis, serving as key enzymes for producing important flavonoids [30,31]. It is hypothesized that higher expression of *SbCHS1* and *SbCHS2* in GL18 and GL74 leads to the accumulation of naringenin, laying the foundation for further flavonoid synthesis. In the first metabolic pathway, naringenin can be converted to delphinidin derivatives, which exhibit blue-purple, via dihydromyricetin. GL18 may accumulate more delphinidin derivatives, while GL21 shows limited production, possibly due to insufficient precursor availability. In the second pathway, naringenin can also be converted to cyanidin derivatives, which display magenta, via dihydroquercetin. Anthocyanin synthase (ANS) [32] and leucoanthocyanidin reductase (LAR) [33], key enzymes in the anthocyanin and proanthocyanidin biosynthesis pathways, compete for the same substrate (leucoanthocyanidin) to produce cyanidin and (+)-catechin, respectively. Previous studies have demonstrated that suppression of *ANS* gene expression significantly impairs anthocyanin biosynthesis in petals, resulting in aberrant pigmentation phenotypes [34]. These findings suggest that *ANS* transcriptional regulation serves as a critical determinant of floral and fruit coloration through its control over anthocyanin accumulation pathways [35]. The *VvMYBPA1* gene family has been shown to specifically induce proanthocyanidin biosynthesis without affecting anthocyanin accumulation [36,37]. In this study, metabolomic profiling revealed that GL18 and GL74 exhibited higher cyanidin accumulation level compared to GL21, resulting in enhanced accumulation of downstream cyanidin derivatives. This differential accumulation pattern may explain the darker grain coloration observed in GL18 and GL74 relative to GL21 [36]. In the third pathway, naringenin undergoes sequential conversion to pelargonidin-3-O-glucoside—a bright red pigment—via aromadendrin, with dihydroflavonol-4-reductase (DFR) serving as the key rate-limiting enzyme [37]. Previous studies demonstrated that *DFR* expression level are significantly elevated in red fruits containing pelargonidin-3-O-glucoside compared to white-fruited varieties [38], suggesting its regulatory role in pigmentation [39]. Notably, while pelargonidin-3-O-glucoside was detected in all sorghum accessions, its accumulation level remained statistically equivalent across accessions, indicating a conserved basal contribution of this pathway to color formation. Collectively, these findings reveal distinct flavonoid metabolic profiles among sorghum accessions with varying grain colors, providing novel insights into the metabolic regulatory networks governing sorghum grain coloration.

### Accession-specific metabolite accumulation patterns underpin differential quality contribution to baijiu

Sorghum has a long history of cultivation in China and is frequently utilized as a primary raw material for brewing [40]. During fermentation, amino acid metabolites play a pivotal role in shaping the flavor profile of alcoholic beverages [41]. For instance, tryptophan can be converted to 4-methylphenol under specific conditions, contributing a distinct cellar mud aroma [42]. Additionally, leucine metabolism generates 2-methyl-1-butanol, which imparts unique onion and malt aromas to distilled spirits [43,44]. In this study, tryptophan and L-leucine were detected at lower accumulation level in GL18 but at higher level in GL21 and GL74, suggesting that GL21 and GL74 may more readily produce metabolites with distinctive aromas and flavors during fermentation compared to GL18. β-Phenylethanol, an important aromatic compound with sweet, rose-like, and honey-like notes, has phenylglucoside as its precursor, which was found in higher accumulation level in GL74 [45]. This implies that GL74 may generate more β-phenylethanol during fermentation, thereby influencing the flavor profile of baijiu. Flavor precursors release volatile flavor compounds, contributing to aroma development [46]. Current research on flavor precursors primarily focuses on glycosides, such as L-rhamnofuranose and D-glucose [46]. In GL21, higher level of glycosides including L-rhamnofuranose, L-rhamno-1,4-lactone, and agnuside were observed, suggesting that it may produce more flavor compounds during fermentation, further enhancing the flavor complexity of baijiu. Furthermore, the abundance of aroma precursors in raw materials significantly influences the flavor profile of baijiu [47,48]. Gas chromatography-mass spectrometry analysis has identified numerous free aromatic compounds, including phenol and thymol [49]. Phenol contributes a medicinal aroma [50], while thymol exhibits spicy and herbal notes [51]. In GL18, phenol and thymol accumulation level were significantly higher than in GL21 and GL74, potentially conferring a unique flavor profile to baijiu. Conversely, benzaldehyde—characterized by almond aroma and slight bitterness [52]—was more abundant in GL21 and GL74 than in GL18. Notably, 2-phenylethanol, 2-hydroxybenzaldehyde, and (±)-furaneol were particularly prominent in GL74. 2-Phenylethanol is a key contributor to rose-like aroma in distilled spirits [53], while 2-hydroxybenzaldehyde imparts burnt and almond-like scents [54]. (±)-Furaneol, known for its intense caramel and fruity aromas, is widely used to enhance sweetness and fruitiness in food and beverages [55]. These metabolites suggest that GL74 may provide a more distinctive aromatic foundation for baijiu. Additionally, hexanal—contributing sweet, floral, grassy, and fruity notes [56]—showed higher accumulation in GL21, indicating its potential to produce a richer flavor profile during fermentation. In summary, the distinct metabolic profiles of these sorghum accessions suggest varying flavor characteristics in the resulting baijiu. This diversity enables targeted selection of specific accessions to achieve desired flavor expressions, thereby enhancing the sensory quality of the final product.

### Sorghum grain metabolic diversity shapes vinegar quality specialization

Sorghum also serves as a critical raw material for vinegar production [57], with its metabolic composition directly influencing the sensory quality of fermented products [58]. Sucrose, mannose, and fructose, as primary energy sources during fermentation [59,60], exhibit varying level that may directly impact the vinegar fermentation process. Additionally, polyols such as sorbitol, xylitol, and mannitol play crucial roles in vinegar production [61]. GL21 and GL74 showed significantly higher relative accumulation level of these carbohydrates compared to GL18, suggesting that GL21 and GL74 may support more robust microbial activity in vinegar production compared to GL18. The observed amino acid profiles further elucidate fermentation-related quality mechanisms. Arginine, a key precursor for melanoidin production via Maillard reactions during vinegar aging [62], showed higher accumulation in GL74, while GL21 was characterized by elevated L-proline levels—a signature amino acid of Shanxi aged vinegar [63]. This metabolic divergence indicates that GL74 may enhance vinegar color intensity through melanoidin formation, whereas GL21 could contribute to the characteristic savory notes of traditional Chinese vinegar. Organic acids are critical flavor components in vinegar [64], with acetic acid and lactic acid being the most abundant. Higher lactic acid accumulation level can impart a sweet acidity to vinegar [65]. In cereal vinegars, quinic acid is a major organic acid [66], while vanillic acid and caffeic acid are prominent phenolic compounds in Shanxi aged vinegar [67]. L-Malic acid, an important intermediate in the tricarboxylic acid cycle [68], and sinapic acid, a major phenolic acid in many vinegars [15], also play significant roles in flavor formation. Specifically, vanillic acid and L-malic acid were more abundant in GL18, lactic acid and caffeic acid were predominant in GL21, and sinapic acid and quinic acid showed higher accumulation in GL74. These findings demonstrate that the metabolic diversity among sorghum accessions directly correlates with specific flavor compound production pathways during fermentation. By linking accessions’ metabolite profiles to fermentation outcomes, this study provides a scientific basis for targeted raw material selection in vinegar production. The distinct metabolic specializations of GL18, GL21, and GL74 enable producers to modulate flavor profiles according to market preferences—whether prioritizing fruity complexity (GL18), mellow richness (GL21), or color intensity with balanced astringency (GL74). This approach aligns with the research objective of developing genotype-dependent fermentation strategies to enhance product diversification in traditional vinegar industries.

## Conclusion

In this study, three sorghum accessions with distinct grain color phenotypes (GL18, GL21, and GL74) were selected for comprehensive metabolomic analysis. The results demonstrated that GL18 exhibited the highest flavonoid accumulation level, followed by GL74 and GL21. This differential accumulation pattern resulted from the coordinated regulation of three flavonoid-related metabolic pathways, which collectively determined the observed grain color variations among accessions. The identified metabolites and their associated biosynthetic processes provide a mechanistic framework for understanding the molecular basis of sorghum grain color diversity. Furthermore, these sorghum accessions, characterized by significantly different metabolite profiles, may serve as distinct raw materials for baijiu and vinegar fermentation, offering tailored options for the production of baijiu and vinegar with unique quality profiles.

## Supporting information

S1 Table KEGG pathway enrichment analysis of differential metabolites in sorghum accessions. (XLSX)
